# A Minimal Nitrogen Fixation Gene Cluster from *Paenibacillus* sp. WLY78 Enables Expression of Active Nitrogenase in *Escherichia coli*

**DOI:** 10.1371/journal.pgen.1003865

**Published:** 2013-10-17

**Authors:** Liying Wang, Lihong Zhang, Zhangzhi Liu, Dehua Zhao, Xiaomeng Liu, Bo Zhang, Jianbo Xie, Yuanyuan Hong, Pengfei Li, Sanfeng Chen, Ray Dixon, Jilun Li

**Affiliations:** 1 State Key Laboratory for Agrobiotechnology and College of Biological Sciences, China Agricultural University, Beijing, P. R. China; 2 College of Life Science, Shanxi Normal University, Linfen, P. R. China; 3 Synthetic Biology Center, Department of Biological Engineering, Massachusetts Institute of Technology, Cambridge, Massachusetts, United States of America; 4 Department of Molecular Microbiology, John Innes Centre, Norwich, United Kingdom; Universidad de Sevilla, Spain

## Abstract

Most biological nitrogen fixation is catalyzed by molybdenum-dependent nitrogenase, an enzyme complex comprising two component proteins that contains three different metalloclusters. Diazotrophs contain a common core of nitrogen fixation *nif* genes that encode the structural subunits of the enzyme and components required to synthesize the metalloclusters. However, the complement of *nif* genes required to enable diazotrophic growth varies significantly amongst nitrogen fixing bacteria and archaea. In this study, we identified a minimal *nif* gene cluster consisting of nine *nif* genes in the genome of *Paenibacillus* sp. WLY78, a gram-positive, facultative anaerobe isolated from the rhizosphere of bamboo. We demonstrate that the *nif* genes in this organism are organized as an operon comprising *nifB*, *nifH*, *nifD*, *nifK*, *nifE*, *nifN*, *nifX*, *hesA* and *nifV* and that the *nif* cluster is under the control of a σ^70^ (σ^A^)-dependent promoter located upstream of *nifB*. To investigate genetic requirements for diazotrophy, we transferred the *Paenibacillus nif* cluster to *Escherichia coli*. The minimal *nif* gene cluster enables synthesis of catalytically active nitrogenase in this host, when expressed either from the native *nifB* promoter or from the T7 promoter. Deletion analysis indicates that in addition to the core *nif* genes, *hesA* plays an important role in nitrogen fixation and is responsive to the availability of molybdenum. Whereas *nif* transcription in *Paenibacillus* is regulated in response to nitrogen availability and by the external oxygen concentration, transcription from the *nifB* promoter is constitutive in *E. coli*, indicating that negative regulation of *nif* transcription is bypassed in the heterologous host. This study demonstrates the potential for engineering nitrogen fixation in a non-nitrogen fixing organism with a minimum set of nine *nif* genes.

## Author Summary

Biological nitrogen fixation plays an essential role in the nitrogen cycle, sustaining agricultural productivity by providing a source of fixed nitrogen for plants and ultimately animals. The enzyme nitrogenase that catalyses the reduction of atmospheric dinitrogen to ammonia contains one of the most complex heterometal cofactors found in biology. Biosynthesis of nitrogenase and provision of support for its activity requires a large number of nitrogen fixation (*nif*) genes, which vary according to the physiological lifestyle of the host organism. In this study, we identified a *nif* cluster with reduced genetic complexity, consisting of nine genes organized as a single operon in the genome of *Paenibacillus* sp. WLY78. When transferred to *Escherichia coli*, the *Paenibacllus nif* cluster enables synthesis of catalytically active nitrogenase, which is competent to reduce both acetylene and dinitrogen as substrates of the enzyme. Environmental regulation of *nif* gene expression in *Paenibacillus*, in response to either oxygen or fixed nitrogen, is circumvented when the *nif* operon is expressed from its native promoter in *E. coli*, suggesting that *nif* transcription in *Paenibacillus* is negatively regulated in response to these effectors.

## Introduction

Although fixed nitrogen plays a critical role in the global food supply, overuse of chemical nitrogen fertilizers has led to increased costs for farmers and harmful consequences for the environment and human health. Biological nitrogen fixation, the conversion of atmospheric N_2_ to NH_3_, offers a natural means of providing nitrogen for plants [1]. There has been a long-standing interest in reducing dependence on fertilizers through engineering non-legume crops that “fix” nitrogen but maintain growth yields [2], [3]. Achieving this goal will require elucidating the minimal number of genes required to sustain biological nitrogen fixation.

Most biological nitrogen fixation is catalyzed by molybdenum-dependent nitrogenase, which is distributed within bacteria and archaea. This enzyme is composed of two component proteins, MoFe protein and Fe protein. The MoFe protein component is an α_2_β_2_ heterotetramer (encoded by *nifD* and *nifK*) that contains two metalloclusters; FeMo-co, a [Mo-7Fe-9S-C-homocitrate] cluster which serves as the active site of substrate binding and reduction and the P-cluster, a [8Fe-7S] cluster which shuttles electrons to FeMo-co. The Fe protein (encoded by *nifH*) is a homodimer bridged by an intersubunit [4Fe-4S] cluster that serves as the obligate electron donor to the MoFe protein. The assembly pathway for the biosynthesis of nitrogenase is complex. Apart from the structural subunits encoded by *nifH*, *nifD* and *nifK*, several genes are required for the biosynthesis of the metalloclusters, in addition to other gene products necessary to produce a fully functional enzyme. It is now well established from genetic and biochemical analysis that *nifE nifN*, *nifX nifB*, *nifQ*, *nifV*, *nifY* and *nifH* contribute to the synthesis and insertion of FeMo-co into nitrogenase, that *nifU nifS* and *nifZ* play an important role in synthesis of metalloclusters and that *nifM* is required for proper folder of nitrogenase Fe protein [4]–[7].

**Figure 1:**
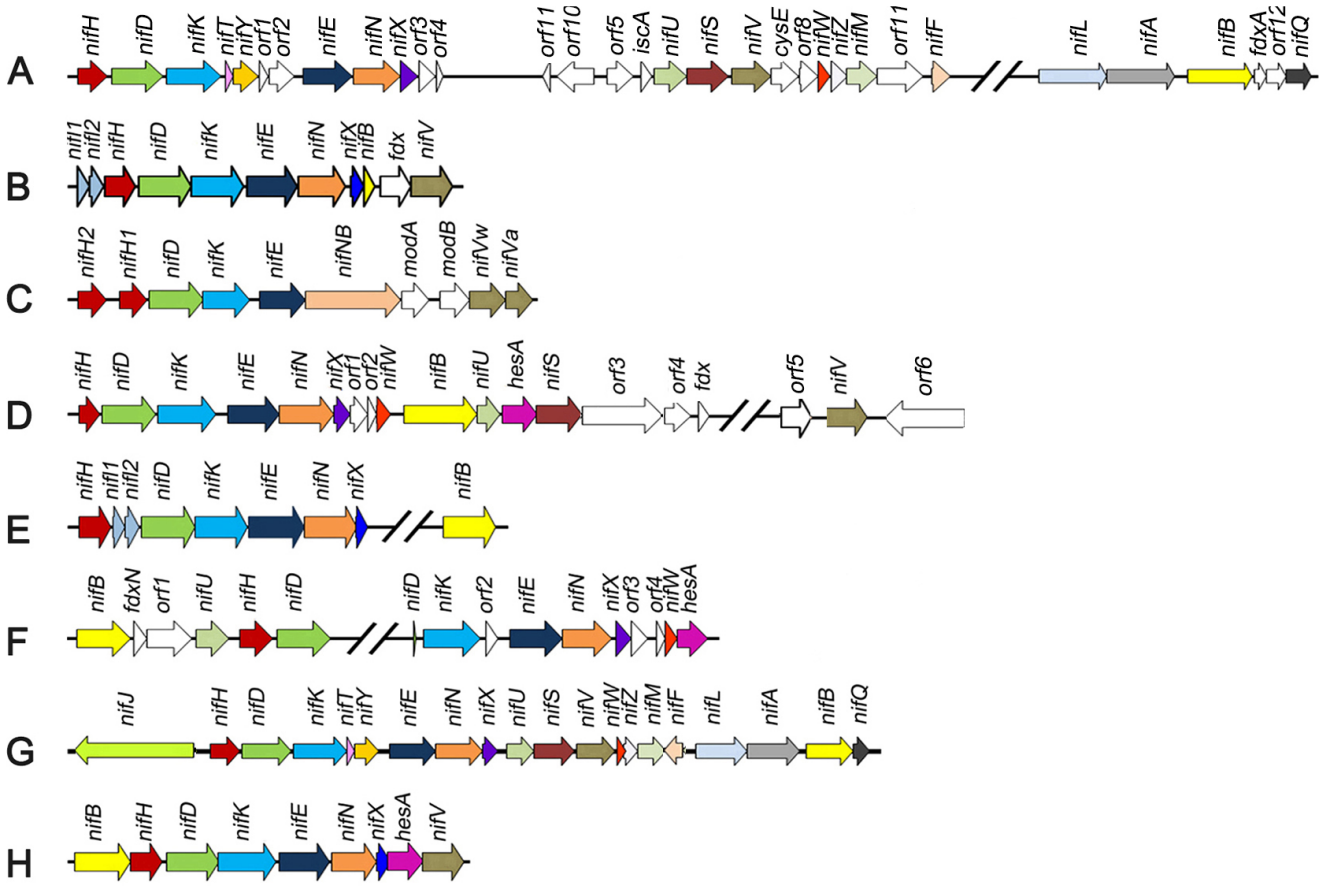
Comparison of the *Paenibacillus* sp. WLY78 *nif* gene cluster with representative clusters from diverse diazotrophic bacteria and archaea. (A) *Azotobacter vinelandii*, (B) *Heliobacterium chlorum*, (C) *Clostridium acetobutylicum* W5, (D) *Frankia sp. EAN1pec*, (E) *Methanococus maripaludis*, (F) *Anabaena variabilis* ATCC 29413, (G) *Klebsiella oxytoca* M5al, (H) *Paenibacillus* sp. WLY78.

The inventory of genes required for diazotrophy varies greatly amongst species, dependent upon the environmental niche and physiology of the host. For example, in *Klebsiella oxytoca*, twenty *nif* genes are co-located within a ∼24 kb cluster [8], whereas in *Azotobacter vinelandii* the *nif* genes are more dispersed and distributed as two clusters in the genome [9] (Figure 1). However, in contrast to these paradigm diazotrophs, other nitrogen fixing organisms possess a more restricted *nif* gene set, for example the archeon, *Methanococcus maripaludis*, contains only 9 *nif* genes (Figure 1), two of which *nifI1* and *nifI2*, are not essential for nitrogen fixation, but serve a regulatory function [10]. Analysis of the distribution of *nif* gene sequences within microbial genomes indicates that nearly all diazotrophs have a minimal gene set consisting of six conserved genes *nifH*, *nifD*, *nifK*, *nifE*, *nifN*, and *nifB* [11]. This concurs with the minimal catalytic core required to assemble FeMo-co *in vitro* [12].

One of the difficulties in determining the precise genetic requirements for nitrogen fixation in diazotrophs arises from the presence of “housekeeping” counterparts in the genome that may substitute for the function of known *nif* genes. This may be particularly important in the case of diazotrophs that possess minimal *nif* gene clusters. One approach to investigate the inventory of genes required for diazotrophy in such cases is to transfer the *nif* cluster to a distantly related organism that does not have the capacity to fix nitrogen. *Escherichia coli* provides an important model organism for such studies as physiology and gene function is extremely well understood. Since transfer of the complete cluster of 20 *nif* genes from *K. oxytoca* to *E. coli* confers the ability to fix nitrogen [13], we were interested to determine whether a more evolutionary distant *nif* gene cluster would also enable nitrogenase activity in *E. coli*. In this study, we identified a minimal *nif* cluster consisting of nine genes, in the genome of *Paenibacillus* sp. WLY78 (Figure 1). The cluster is apparently transcribed from a single σ^A^ (σ^70^)-like promoter that functions in *E. coli* to express active nitrogenase. Our results may have important implications for future engineering of nitrogen fixation in non-diazotrophs.

## Results

### Genome sequencing of *Paenibacillus* sp. WLY78 identifies a minimal nitrogen fixation (*nif*) gene cluster

*Paenibacillus* sp. WLY78 is a gram-positive, facultative anaerobic, endospore-forming bacterium isolated from the rhizosphere of bamboo [14]. This bacterium has potential use in agriculture, since it is able to fix nitrogen and also produces antimicrobial substances. We therefore determined the genome sequence of this organism and identified a nitrogen fixation gene cluster consisting of nine genes arranged within a 10.5 kb region in the order, *nifB*, *nifH*, *nifD*, *nifK*, *nifE*, *nifN*, *nifX*, *hesA* and *nifV* (Figure 1). The *nif* cluster is flanked by genes coding for a hypothetical protein upstream and an ABC transporter downstream. The G+C content of this *nif* cluster was higher than the average of the entire genome (52.8% vs. 45.1%), suggesting that it may have been acquired by horizontal gene transfer. The *Paenibacillus* sp. WLY78 *nif* cluster is one of the most compact compared with other dizaotrophs described to date (Figure 1). Similar *nif* gene arrangements and neighborhoods are observed in other *Paenibacillus* strains, including *Paenibacillus terrae* HPL-003. Multiple alignments revealed that the predicted protein products of the *Paenibacillus nif* genes showed 67–80% identity to the corresponding *nif* gene products of their gram-positive counterparts [15], but showed only 35–69% identity to the corresponding *nif* genes of *K. oxytoca* and *A. vinelandii* (Table S1). The gene designated as *hesA*, which is located between *nifX* and *nifV* is found in other *nif* clusters (Figure 1) and the predicted product shares ∼45% identity with the putative molybdenum cofactor biosynthesis protein HesA of *Frankia alni* ACN14a [16] and *Cyanothece* sp. ATCC 51142 [17]. HesA is a member of the ThiF-MoeB-HesA family and contains an N-terminal nucleotide binding domain and a C-terminal MoeZ/MoeB-like domain.

**Figure 2:**
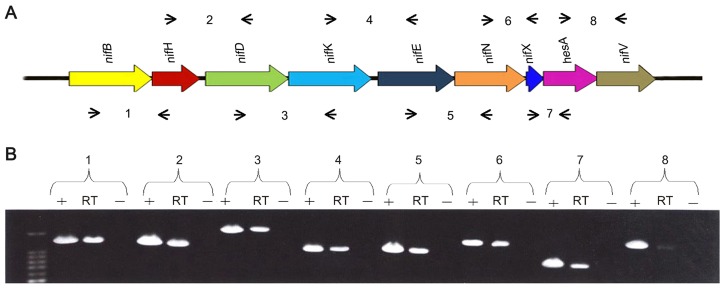
The *nif* genes of *Paenibacillus* sp. WLY78 are organized in an operon as determined by RT-PCR. (A) Outline of the strategy. Primers used and amplified products (numbered) are given below the schematic representation of the genes. (B) Result of RT-PCR reactions with RNA from *Paenibacillus* sp. WLY78 grown under N_2_-fixing conditions. The numbering on the top of the gels corresponds to the product numbers drawn schematically in the outline given above. RT, standard RT-PCR reaction; (*−*), negative control in which no reverse transcriptase was added to the RT reaction; (*+*), positive control in which genomic DNA was used as template in the RT-PCR.

RT-PCR experiments using primers designed to span across intergenic regions indicated that the nine genes within the *nif* cluster are organized in a single operon (Figure 2). Single operon *nif* clusters have been reported in gram-positive prokaryotes and in the archaea, e.g. *Heliobacterium chlorum* [18] and *Methanococcus maripaludis* [19]. However, in contrast to these *nif* clusters *Paenibacillus* sp. WLY78 does not contain the negative regulatory genes *nifI1* and *nifI2* (homologues of *glnB*), which are involved in post-translational regulation of nitrogenase activity in response to fixed nitrogen [10].

### Characterization of the *Paenibacillus* sp. WLY78 *nif* promoter and transcription unit

**Figure 3:**
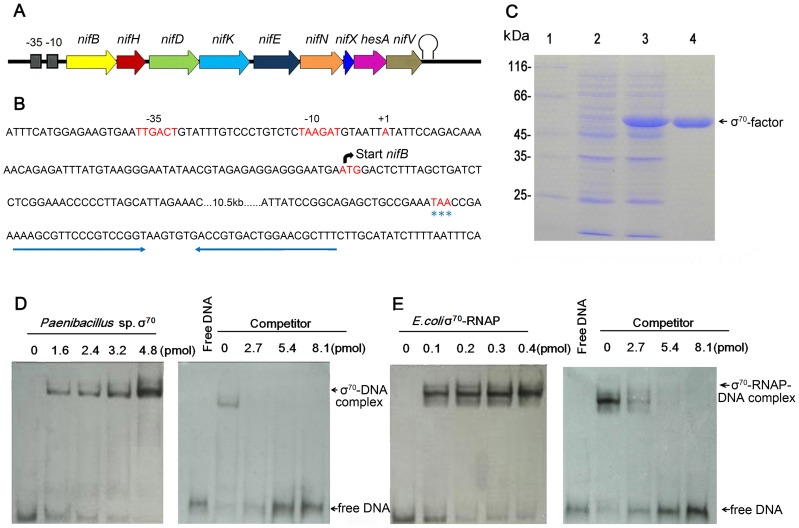
Characterization of the *nif* promoter of *Paenibacillus* sp. WLY78. (A) Schematic representation of the *Paenibacillus* sp. WLY78 *nif* operon. (B) Nucleotide sequence of the *nifB* promoter and the putative terminator sequence flanking the 3′ end of *nifV*. The asterisks below TAA indicate the *nifV* stop codon. (C) Overexpression and purification of σ^70^ from *Paenibacillus* sp. WLY78. Lane 1: protein marker; lane 2: uninduced protein; lane 3: induced protein; lanes 4: purified σ^70^ factor. (D) Electrophoretic mobility shift assays (EMSA) demonstrating binding of *Paenibacillus* σ^70^ to the 50 bp *nifB* promoter DNA fragment (final concentration 0.03 pmol). The protein concentration is indicated in pmol above each lane (left hand panel). In the right hand panel, the protein concentration was maintained at 2.4 pmol and unlabeled *nifB* promoter fragment was added as competitor (concentration indicated above each lane). (E) EMSA experiments demonstrating binding of *E. coli* σ^70^-RNAP to the 50 bp *nifB* promoter DNA fragment (final concentration 0.03 pmol). The protein concentration is indicated in pmol above each lane (left hand panel). In the right hand panel, the protein concentration was maintained at 0.2 pmol and unlabeled *nifB* promoter fragment was added as competitor (concentration indicated above each lane).

The transcriptional start site (TSS) of the *nif* gene cluster in *Paenibacillus* sp. WLY78 was determined by using the 5′-RACE (Rapid Amplification of cDNA Ends) method. The TSS was located 59 bp upstream of the translational start site of *nifB* and a putative promoter was identified 6 nucleotides preceding the TSS (Figure 3). The −35 (TTGACT) and −10 (TAAGAT) sequences in the *nifB* promoter were similar to the corresponding consensus sequences (TTGACA and TATAAT respectively) of *E. coli* σ^70^-dependent promoters. Unlike other members of the Bacillales, the *Paenibacillus* sp. WLY78 genome does not contain a homolog of *rpoN* and consequently σ^54^-dependent −24/−12 promoter sequences were not observed either upstream of the *nif* cluster or in the 5′ regions of other genes in the *Paenibacillus* sp. WLY78 genome (data not shown). Downstream of *nifV*, a potential transcriptional termination site was identified, containing two potential stem loops followed by a T-rich region (Figure 3B). These findings indicate that the *nif* genes in *Paenibacillus* sp. WLY78 are organized as a single operon containing 9 genes, which is transcribed from an *rpoD*-dependent promoter.

To analyze the σ^70^-dependentcy of the *nifB* promoter, electrophoretic mobility shift assays (EMSA) were carried out using either *E. coli* σ^70^-RNAP (RNA polymerase) or σ^70^ from *Paenibacillus* sp. WLY78, which was overexpressed and purified from *E. coli* (Figure 3C). EMSA experiments revealed that both purified σ^70^ from *Paenibacillus* sp. WLY78 and *E. coli* σ^70^-RNAP holoenzyme bind to the 50 bp *nifB* promoter fragment. Competition experiments with non-labelled *nifB* DNA indicated that the *E. coli* RNAP holoenzyme binds more tightly to this DNA fragment, since higher concentrations of competitor were apparently required to dissociate the *E. coli* σ^70^-RNAP (Figure 3, panels D and E). EMSA experiments with a scrambled double-stranded oligonucleotide did not reveal binding of either protein (data not shown). These results are consistent with the ability of σ^A^ (σ^70^) of *Bacillus subtilis* to bind to promoters independent of core RNAP [20], [21].

**Figure 4:**
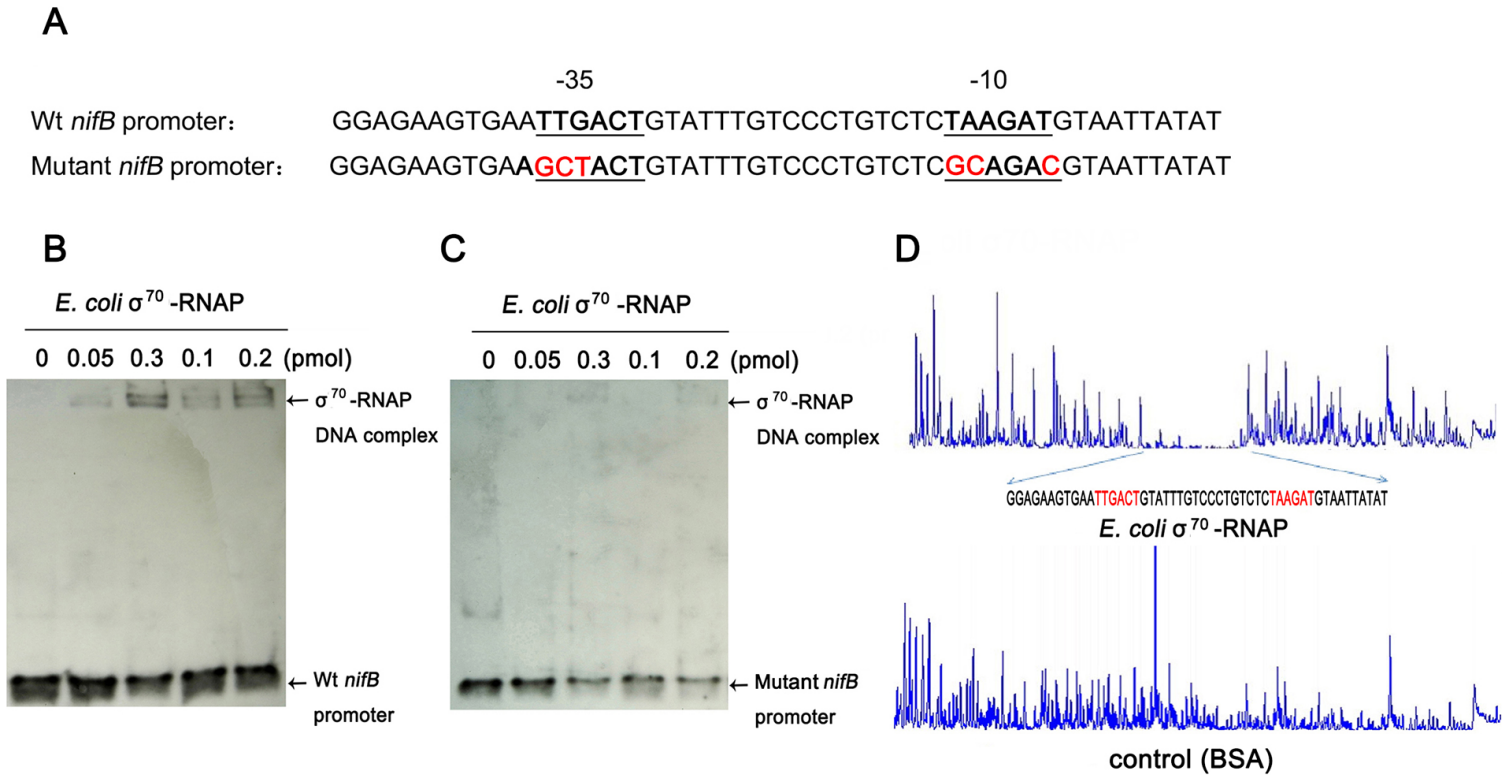
*E. coli* σ^70^-RNAP binds preferentially to the −35 region and −10 region of the *nifB* promoter of *Paenibacillus* sp. WLY78. (A) Substitutions introduced in the *nifB* promoter sequence. The sequences of the −35 and −10 regions of the *nifB* promoter are underlined (Wt indicates the wild-type sequence). Base substitutions in the mutant promoter are indicated in red. (B) and (C) EMSA experiments comparing the binding of *E. coli* σ^70^-RNAP to the wild-type *nifB* promoter fragment (panel B) with the mutant promoter fragment (panel C). The protein concentration is indicated above each lane. (D) DNase I footprinting of the interaction of *E. coli* σ^70^-RNAP with the *nifB* promoter using an automated capillary sequencer. The top lane is an electropherogram obtained in the presence of σ^70^-RNAP with the sequence protected from cleavage shown below. A control electropherogram obtained from a reaction containing BSA is shown in the bottom lane.

To further examine the specificity of binding of *E. coli* σ^70^-RNAP to the *Paenibacillus* sp. WLY78 *nifB* promoter, we made substitutions in the −35 (TTGACT to **GCT**ACT) and −10 (TAAGAT to **GC**AGA**C** ) regions of the promoter (Figure 4A). Binding of *E. coli* σ^70^-RNAP to the *nifB* promoter fragment was weakened considerably by the presence of the −35 and −10 substitutions (compare Figure 4, panels B and C), suggesting that *E. coli* σ^70^-RNAP specifically interacts with the *nifB* promoter from *Paenibacillus* sp. WLY78. In order to confirm this, we performed DNAse I footprinting with a fluorescently labeled 319 bp DNA target carrying the *nifB* promoter and analyzed the digested DNA fragments using a capillary sequencer. As expected, the region protected from DNAse I digestion corresponded to the *nifB* promoter, confirming that *E. coli* σ^70^-RNAP specifically binds to the −35 and −10 regions upstream of the transcription start site. (Figure 4D). Our studies thus demonstrate that the *nifB* promoter of *Paenibacillus* sp. WLY78 is σ^70^-dependent and thus distinct from the typical σ^54^-dependent −24/−12 promoters found upstream of *nif* genes in gram-negative diazotrophs.

**Figure 5:**
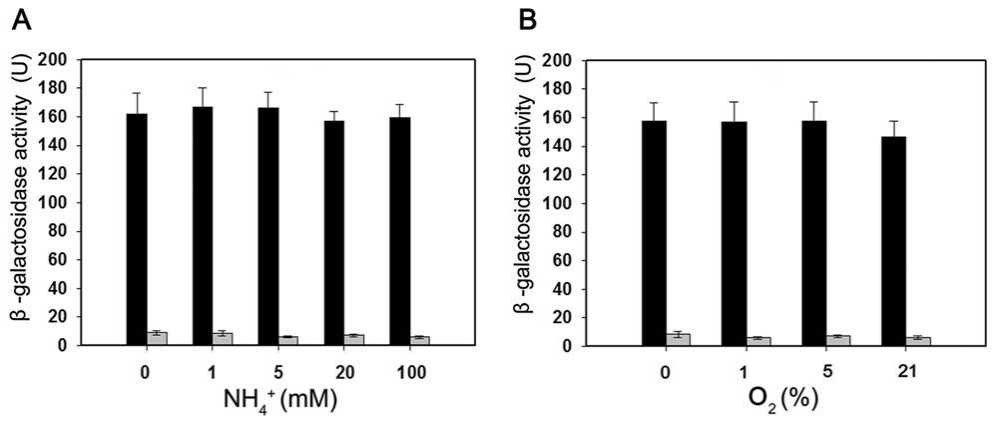
Expression of the *Paenibacillus* sp. WLY78 P*nif*B::lacZ promoter fusion is constitutive in *E. coli*. Black bars indicate expression of ß-galactosidase driven by the *nifB* promoter; grey bars indicate the level of ß-galactosidase activity exhibited by the vector plasmid (pPR9TT) alone. Cultures were grown in nitrogen deficient medium, with 2 mM glutamate as nitrogen source, either anaerobically with the indicated concentrations of NH_4_Cl (left panel) or with the indicated initial oxygen concentrations shown in the right-hand panel. Error bars indicate the standard deviation observed from at least two independent experiments.

To verify if the *nifB* promoter of *Paenibacillus* sp. WLY78 is functional in *E. coli*, it was fused to the *lacZ* reporter gene. The level of β-galactosidase activity expressed from the *PnifB::lacZ* fusion in *E. coli* strain JM109 was not influenced either by the concentration of fixed nitrogen in the culture medium or by the external oxygen concentration (Figure 5). Hence, the *Paenibacillus* sp. WLY78 *nifB* promoter is apparently recognized by *E. coli* σ^70^ RNA polymerase *in vivo*. These data concur with previous studies where promoters of gram-positive bacteria, for example, *Bacillus stereothermophilus* [22] and *Corynebacterium glutamicum* [23], were shown to be functional in *E. coli*.

### The *Paenibacillus nif* gene cluster enables nitrogen fixation by *E. coli*

**Figure 6:**
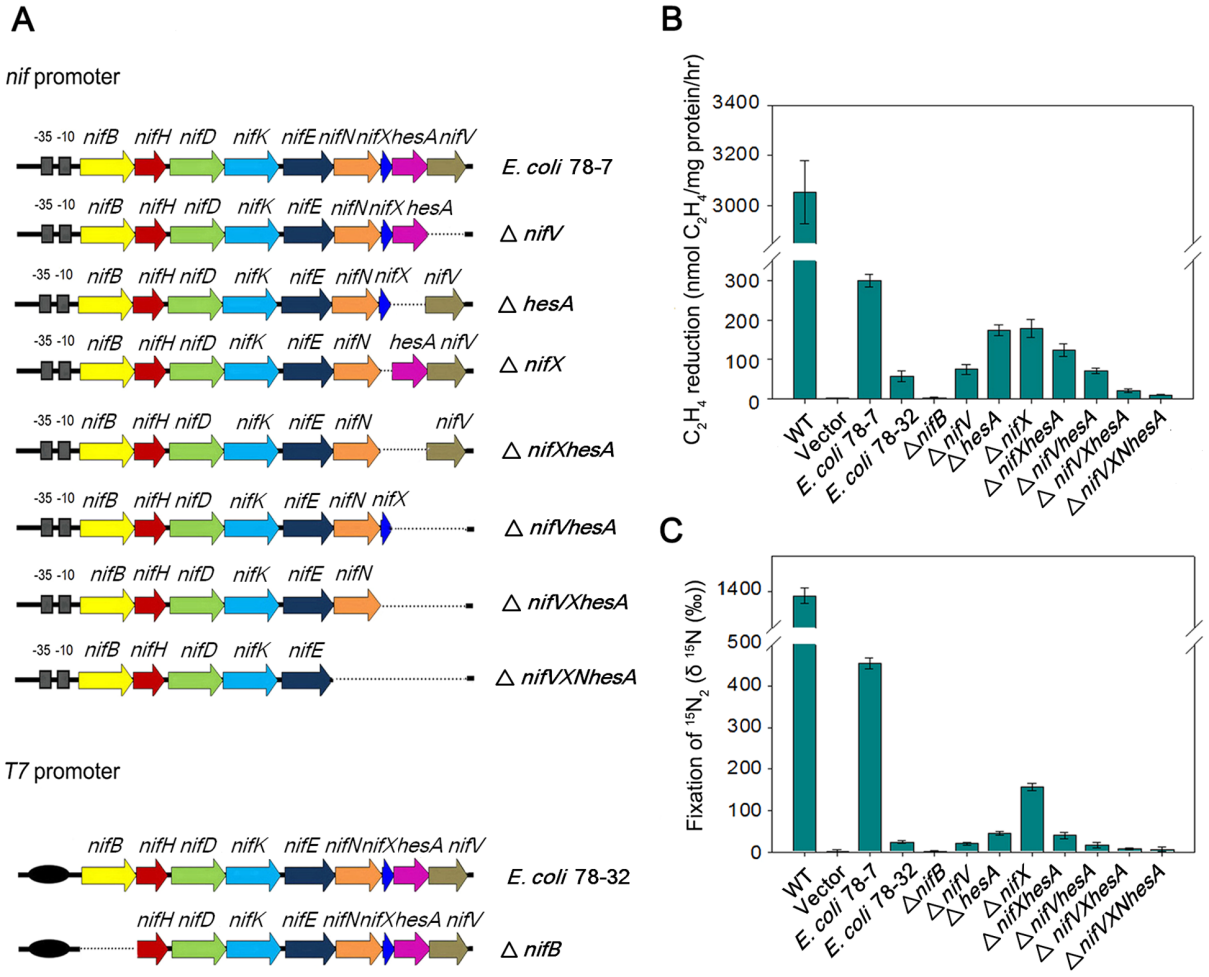
Engineered E. coli strains and their nitrogen fixation abilities. (A) Scheme showing the genetic organization of the engineered *E. coli* strains. (B) and (C) Nitrogenase activities of engineered strains and their deletion variants compared with *Paenibacillus* sp. WLY78 (bars marked as “WT”) and *E. coli* JM109 carrying the empty vector plasmid pHY300PLK (bars marked as “vector”). Strains were grown anaerobically in nitrogen-deficient conditions and the cultures were assayed either for acetylene reduction (panel B) or for ^15^N_2_ incorporation (panel C). Error bars indicate the standard deviation observed from at least two independent experiments.

To transfer the *Paenibacillus nif* gene cluster to *E. coli*, we cloned a 10.5-kb DNA fragment (containing the sequence from the ATG start codon of *nifB* to the TAA stop codon of *nifV*) in the expression vector pET-28b bringing the *nif* genes under control of the T7 promoter. This construct was then transformed into *E. coli* BL21 (DE3), yielding the engineered *E. coli* strain 78-32. We further cloned the 11-kb full-length *nif* cluster containing its own *nif* promoter and the contiguous nine genes *nifBHDKENXhesAnifV* into the multicopy plasmid pHY300PLK and transformed this into *E. coli* JM109, yielding the engineered *E. coli* strain 78-7 (Figure 6A). To determine whether the *Paenibacillus nif* cluster functions in *E. coli*, we employed two independent methods to assess nitrogenase activity; firstly, reduction of the alternative substrate acetylene to ethylene, which can be readily quantified by gas chromatography [24], [25] and secondly, a ^15^N_2_ enrichment assay to directly measure the incorporation of this tracer into organic nitrogen [26]. When grown anaerobically in nitrogen-deficient medium, *Paenibacillus* sp. WLY78 exhibits both acetylene reduction and ^15^N_2_ incorporation (Figure 6, panels B and C). The engineered *E. coli* strain 78-7, which expresses the *nif* genes from the native promoter showed approximately 10% of the specific activity for acetylene reduction when compared with *Paenibacillus* and was competent to assimilate ^15^N_2_. In contrast, when expressed from the T7 promoter and induced with 2 mM IPTG the *Paenibacillus nif* cluster exhibited relatively low levels of nitrogenase activity in the recombinant *E. coli* strain 78-32 (Figure 6). Therefore, the engineered *E. coli* strain 78-7 was used for most of the studies reported here. When compared with the recipient *E. coli* strain JM109, the engineered strain 78-7 had an identical cellular phenotype when analyzed by Biolog phenotypic microarrays [27] (data not shown). In comparison with the *Paenibacillus* sp. WLY78 strain, which is capable of diazotrophic growth, the engineered *E. coli* strain 78-7 grew poorly in liquid media with dinitrogen as the sole nitrogen source (data not shown). Therefore, although the recombinant strain expresses active nitrogenase and assimilates ^15^N_2_, this does not enable the engineered *E. coli* strain to grow as a diazotroph.

### Minimal *Paenibacillus nif* genes required for nitrogenase activity

To further determine the minimal *nif* genes required for nitrogen fixation, we constructed a series of *nif* gene deletions (Figure 6). Neither acetylene nor ^15^N_2_ incorporation was detectable in the *nifB* deletion, supporting the original observation that *nifB* is essential for synthesis of nitrogenase [5]. When *nifV* was deleted, ^15^N_2_ assimilation decreased more significantly than acetylene reduction, in agreement with the substrate reduction properties of *nifV* mutants [28], which are unable to synthesize the homocitrate moiety of FeMo-co [29]. Deletion of *hesA* also influenced ^15^N_2_ incorporation more significantly than acetylene reduction, suggesting that *hesA* is required for nitrogen fixation. In contrast, deleting *nifX* gave rise to a similar decrease (∼50%) in the reduction of both substrates. In the Δ*nifXhesA* double deletion, nitrogenase activity was similar to that in the single *hesA* mutant, whereas in the double Δ*hesAnifV* deletion, activities were similar to those exhibited by the single *nifV* mutant. Deletion of three (*nifXhesAnifV*) or four genes (*nifNXhesAnifV*) ablated nitrogenase activity. In all cases the phenotypic defects exhibited by the deletions could be reversed by complementation with plasmids bearing the missing genes (data not shown). These results suggest that all nine *Paenibacillus* genes (*nifBHDKENXhesAnifV*) are necessary for optimal nitrogenase activity in *E. coli*.

### Effects of fixed nitrogen and oxygen on *nif* transcription

**Figure 7:**
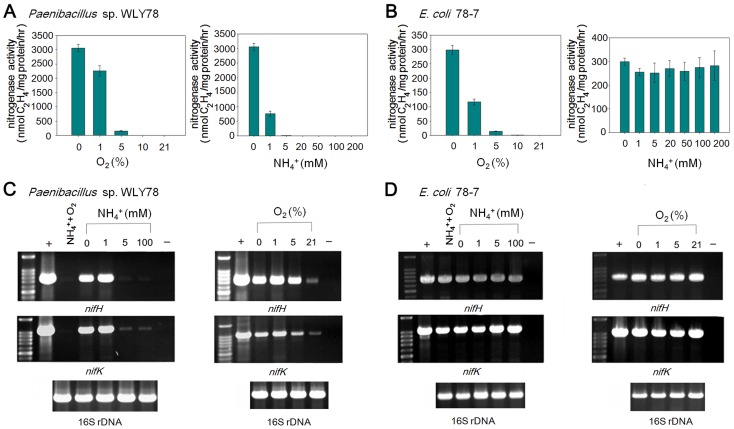
Effects of O_2_ and NH_4_^+^ on nitrogenase activity and *nif* gene transcription. (A) and (B) Comparison of the acetylene reduction activities of *Paenibacillus* sp. WLY78 (panel A) and the engineered *E. coli* 78-7 strain (panel B), when cultures are grown in the presence of either oxygen or ammonium (at the initial concentrations shown on the y axis). Error bars indicate the standard deviation observed from at least two independent experiments. (C) and (D) Comparison of transcription of *nifH* and *nifK* as determined by RT-PCR in *Paenibacillus* sp. WLY78 (panel C) and *E. coli* 78-7 (panel D). Initial concentrations of ammonium and oxygen are indicated above relevant lanes. Lanes labeled “NH_4_^+^,O_2_” indicate that both 2 mM ammonium and 21% oxygen were present. Lanes labeled “+” indicate positive controls in which genomic DNA was used as template in the RT-PCR. Lanes labeled “*−*” indicate negative controls in which no reverse transcriptase was added to the RT-PCR reaction. In each case a parallel RT-PCR reaction was performed to detect the level of 16S rRNA, to provide a loading control (shown beneath relevant lanes).

In many diazotrophs such as *K. oxytoca* and *A. vinelandii*, expression of the *nif* genes is tightly controlled at the transcriptional level in response to the concentration of fixed nitrogen and the oxygen [30]. In addition, the activity of nitrogenase itself can be regulated at the post-translational level in response to environmental effectors [31]. To examine whether the *Paenibacllus nif* cluster is subject to similar regulation, we compared the effects of NH_4_^+^ and O_2_ on in vivo nitrogenase activity and *nif* gene transcription in the native *Paenibacillus* sp. WLY78 strain with that of engineered *E. coli* 78-7 (Figure 7). Both *Paenibacillus* sp. WLY78 and the engineered *E. coli* 78-7 strain did not exhibit nitrogenase activity at O_2_ concentrations above 5% (Figure 7A). In addition, acetylene reduction by *Paenibacillus* sp. WLY78 was not observed at NH_4_^+^ concentrations above 1 mM. In contrast, the engineered *E. coli* strain 78-7 exhibited nitrogenase activity even in the presence of 200 mM NH_4_Cl (Figure 7B). The latter observation suggests that the *Paenibacillus nif* cluster is not subject to regulation by fixed nitrogen in *E. coli*. In agreement with the acetylene reduction data, the α and ß subunits of the MoFe protein and the Fe protein component of nitrogenase were only detectable by Western blotting in *Paenibacillus* sp. WLY78 grown under nitrogen fixation conditions, whereas nitrogenase components were detectable in the engineered *E. coli* strain even in the presence of oxygen (Figure S1).

The influence of oxygen and fixed nitrogen on transcription was assessed by RT-PCR using *nifH* and *nifK* probes. Conversant with the acetylene reduction data, *nif* transcription in *Paenibacillus* sp. WLY78 was inhibited by NH_4_^+^ concentrations above 1 mM and by the presence of 21% oxygen (Figure 7C). In contrast, *nifH* and *nifK* transcription in *E. coli* 78-7 was insensitive to the presence of oxygen and fixed nitrogen (Figure 7D). Thus the *Paenibacillus nif* genes are constitutively transcribed in the engineered strain indicating that the transcriptional regulation observed in the native host does not occur in *E. coli*.

## Discussion

Although the biochemical properties and structure of molybdenum nitrogenases are remarkably similar when purified from diverse bacteria and archaea, genetic requirements for the synthesis and assembly of the enzyme and maintenance of its activity differ widely amongst diazotrophs [11], [32], [33]. Some of this diversity is undoubtedly determined by the environmental lifestyle of each diazotroph, the need to protect the enzyme from damage by oxygen and the requirement to provide sufficient ATP and reductant to support enzyme activity under different physiological conditions. Although the conserved nature of the structural genes and the assembly pathway for FeMoco biosynthesis dictates the presence of a common core of *nif* genes, other functions may be provided by protein counterparts encoded elsewhere in the genome. Alternatively, the large *nif* gene clusters found primarily in Proteobacteria may have evolved from more simple clusters in which assembly, processing and maintenance of nitrogenase activity is less well optimized.

In contrast with earlier studies in which transfer of the complete complement of 20 *nif* genes from *K. oxytoca* enabled *E. coli* to fix nitrogen [12], our results with *Paenibacillus* sp. WLY78 demonstrate that only nine *nif* genes are needed to synthesize active nitrogenase in *E. coli*. The specific activity of the enzyme expressed in *E. coli* was approximately 10% of that observed in *Paenibacillus*, but nevertheless sufficient to provide ^15^N_2_ assimilation. However, synthesis of active nitrogenase in the recombinant *E. coli* strain did not enable diazotrophic growth. This implies that this level of enzyme activity is insufficient to support growth on dinitrogen as sole nitrogen source. However, we cannot rule out the possibility that other physiological factors in *E. coli*, for example the ability to synthesis high levels of nitrogenase proteins under conditions of nitrogen starvation, limit the capacity for diazotrophic growth. Considering the physiological background of *E. coli*, one of the notable absences in the minimal *Paenibacillus nif* gene cluster is the presence of *nifF* and *nifJ*, which provide the electron transport chain to nitrogenase in some diazotrophs [34], [35]. The activity of *Paenibacillus* nitrogenase is therefore likely to be reductant limited in *E. coli* in the absence of this electron transport chain. Another notable absence is *nifM*, which encodes a cis-trans peptidyl prolyl isomerase required for proper folding of nitrogenase Fe protein in diazotrophic proteobacteria [6]. Potentially this function is provided by a counterpart enzyme encoded elsewhere in the genome in other diazotrophs such as *Paenibacillus*. However, a functional equivalent of *nifM* is not present in *E. coli*, since assembly of active *K. oxytoca* Fe protein in this background requires the presence of both *nifH* and *nifM* [36]. The *Paenibacillus* NifH sequence contains the seven conserved proline residues identified in other NifH sequences that are considered to be potential substrates for NifM [6]. However, it is possible that other amino acid substitutions in NifH may enable assembly of Fe protein in the absence of NifM. The *Paenibacillus* sp. WLY78 *nif* gene operon does not contain homologs of the nitrogen fixation-specific iron-sulphur cluster assembly pathway encoded by *nifU* and *nifS*. As in the case of other diazotrophs, this function may be provided by the Suf system, encoded elsewhere in the *Paenibacillus* genome. When *nifH* and *nifM* are expressed in *E. coli*, assembly of the 4Fe-4S cluster in the *K. oxytoca* Fe protein does not require *nifU* and *nifS* [36], [37]. This function is probably provided by the general Isc, Csd or Suf machineries for iron-sulphur cluster biosynthesis in *E. coli*. However, *K. oxytoca nifS* is apparently required for the biosynthesis of the P cluster in the MoFe protein, when Nif polypetides are expressed in *E. coli* [38]. Although *nifU* and *nifS* also participate in FeMoco biosynthesis [37], the requirement for these genes is not absolute, particularly if *nifB* is strongly expressed [38].

Systematic deletion of genes in the *Paenibacillus nif* gene cluster suggests they have functions similar to those of other diazotrophs. As anticipated, *nifB* is essential for nitrogen fixation in *E. coli* and the substrate reduction profile of the *nifV* deletion is expected for a mutant lacking homocitrate synthase and therefore unable to make the homocitrate moiety of FeMo-co [39]. The co-localisation of *hesA* within the *nif* operon is an interesting feature of *Paenibacillus* and other minimal *nif* clusters such as those of cyanobacteria and Frankia (Figure 1). Our deletion analysis demonstrates that *hesA* is important for nitrogenase activity, but the function of *hesA* in nitrogen fixation has not so far been determined. Well-characterized homologs belonging to the ThiF-MoeB-HesA family engage in an ATP-dependent process that activates the C-terminus of partner ubiquitin-like proteins by forming an acyl adenylate complex that facilitates sulfur transfer [40], [41]. Ubiquitin-like proteins contain a conserved C-terminal Gly-Gly motif that is the target for adenylylation by the activating enzyme [42]. Intriguingly, both NifB and NifN from *Paenibacillus* contain C-terminal Gly-Gly motifs and therefore are potential targets for adenylylation by HesA. Given the potential role of HesA as an activating enzyme for sulphur transfer, it is tempting to speculate that HesA may perform a role in metallocluster biosynthesis.

In the Proteobacteria, *nif* genes are generally transcribed from σ^54^-dependent promoters that are subject to transcriptional activation by the enhancer binding protein NifA and are regulated in response to fixed nitrogen and oxygen [30]. However, much less in known about *nif* gene regulation in other diazotrophs where this paradigm is absent. Our results demonstrate that the *nif* cluster of *Paenibacillus* sp. WLY78 is transcribed from a σ^70^-dependent promoter, most likely as a single operon, and that transcription of the *nif* genes is subject to regulation in response to the extracellular concentration of oxygen and fixed nitrogen in *Paenibacillus*. As no transcriptional regulation by either oxygen or fixed nitrogen was detectable when the *Paenibacillus* sp. WLY78 *nif* cluster was expressed from the native *nifB* promoter in *E. coli*, it seems likely that the transcriptional regulation of the *nif* system in *Paenibacillus* involves repression mechanisms. Potential candidates for repression of transcription in response to the nitrogen source are the global nitrogen regulators GlnR and TnrA, which are present in *Paenibacillus* [43].

In summary our results demonstrate that a minimal *nif* gene cluster derived from a gram-positive bacterium can function to synthesize active nitrogenase when expressed in the very different host environment of *E. coli*. This raises various questions concerning the repertoire of genes required for nitrogen fixation and may have important biotechnological implications for engineering diazotophic eukaryotes.

## Materials and Methods

### Strains and media

*Paenibacillus* sp. WLY78 was isolated from the rhizosphere of bamboo in Beijing, China by enrichment in nitrogen-free medium after heating at 100°C for 10 min [14]. Strain WLY78 is similar to *P. polymyxa* based on 16S rDNA phylogeny and whole genome sequencing. *E. coli* strains JM109 and BL21 were used as the recipient strains for constructing the engineered *E. coli* strains carrying nitrogen fixation genes.

*Paenibacillus* sp. WLY78 and the engineered *E. coli* strains were routinely grown in LB or LD medium (per liter contains: 2.5 g NaCl, 5 g yeast and 10 g tryptone) at 30°C with shaking. When appropriate, antibiotics were added in the following concentrations: 40 µg/ml chloramphenicol, 100 µg/ml ampicillin, and 20 µg/ml tetracycline for maintenance of plasmids.

Nitrogen-free, nitrogen-deficient and nitrogen-excess media were used in this study. Nitrogen-free medium contained (per liter) 10.4 g Na_2_HPO_4_, 3.4 g KH_2_PO_4_, 26 mg CaCl_2_• 2H_2_O, 30 mg MgSO_4_, 0.3 mg MnSO_4_, 36 mg Ferric citrate, 7.6 mg Na_2_MoO_4_·2H_2_O, 10 µg p-aminobenzoic acid, 5 µg biotin and 4 g glucose as carbon source. Nitrogen-deficient medium contained 2 mM glutamate as nitrogen source in nitrogen-free medium. Nitrogen-excess medium contained 100 mM NH_4_Cl in nitrogen-free medium [14].

### Acetylene reduction assays

For nitrogenase activity assays, *Paenibacillus* sp.WLY78 and the engineered *E. coli* strains were grown in 5 ml of LD media (supplemented with antibiotics) in 50 ml flasks shaken at 250 rpm for 16 h at 30°C. The cultures were collected by centrifugation, washed three times with sterilized water and then resuspended in nitrogen-deficient medium containing 2 mM glutamate as nitrogen source (supplemented with antibiotics for the engineered *E. coli* strains and IPTG when necessary) to a final OD_600_ of 0.2–0.4. Then, 1 ml of the culture was transferred to a 25-ml test tube and the test tube was sealed with robber stopper. The headspace in the tube was then evacuated and replaced with argon gas [14]. After incubating the cultures for 6–8 h at 30°C with shaking at 250 rpm, C_2_H_2_ (10% of the headspace volume) was injected into the test tubes. After incubating the cultures for a further 3 h, 100 µl of culture headspace was withdrawn through the rubber stopper with a gas tight syringe and manually injected into a HP6890 gas chromatograph to quantify ethylene production. All treatments were in three replicates and all the experiments were repeated three or more times.

For measuring the effect of ammonium on nitrogenase activity, nitrogen-deficient medium was supplemented with NH_4_Cl at the concentrations indicated and the cultures were also grown under anaerobic conditions. For measuring the effect of oxygen on nitrogenase activity, nitrogen-deficient medium containing 2 mM glutamate as nitrogen source was used, and oxygen was adjusted to the initial concentration indicated at the start of the incubation.

### ^15^N_2_ incorporation assay

*Paenibacillus* sp.WLY78 and the engineered *E. coli* strains were grown overnight in LD medium. The cultures were collected and resuspended in 70 ml nitrogen-deficient medium containing 2 mM glutamate as nitrogen source, to an OD_600_ of 0.4 in a 120 ml serum bottle. The serum bottles were filled with N_2_ gas, and then 8 ml gas was removed and 5 ml ^15^N_2_ (99%+, Shanghai Engineering Research Center for Stable Isotope) gas was injected. After 72 h of incubation at 30°C, the cultures were collected, and were freeze dried, ground, weighed and sealed into tin capsules. Isotope ratios are expressed as δ^15^N whose values are a linear transform of the isotope ratios ^15^N/^14^N, representing the per mille difference between the isotope ratios in a sample and in atmospheric N_2_ [26].

### Genome sequencing, assembly and annotation

Total DNA was extracted from *Paenibacillus* sp. WLY78. DNA sequencing was performed using Illumina technologies. A total length of 600,000,120 base pairs of reads was obtained, to enable the assembly of all tags using SOAP denovov. 1.04 assembler [44]. Finally, 87 scaffolds were assembled, giving 101.3-fold coverage of the genome. Glimmer 3 (version 3.0.2) was used for gene finding [45]. Transfer RNA genes were identified by the program tRNAscan-SE [46]. Genes coding for proteins with known functions were annotated by searches against KEGG Genes, Pfam, and SWISSPROT. The complete genome sequence of *Paenibacillus* sp. WLY78 has been deposited at DDBJ/EMBL/Genbank under the accession ALJV00000000. The version described in this paper is version ALJV01000000.

### Construction of recombinant plasmids and recombinant *E. coli* strains

Genomic DNA of *Paenibacillus* sp. WLY78 was used as template for cloning *nif* genes. Primers used for construction of the engineered *E. coli* strains are listed in Table S2. Recombinant plasmids and strains are listed in Table S3.

### Transcription start site identification

The 5′-RACE method was used to determine the transcription start site (TSS) using the SMARTer RACE cDNA Amplification Kit (Clontech). Gene-specific primers are listed in Table S2. The PCR product was cloned into the pMD18-T Vector and then sequenced.

### Overexpression and purification of σ^70^ from *Paenibacillu*s sp. WLY78 in *E. coli*

A 1134 bp DNA fragment carrying the *rpoD* gene (encoding σ^70^ of *Paenibacillus* sp. WLY78) was PCR amplified with primers sigma A-F and sigma A-R (Table S2). The PCR product was ligated to the pET-28b expression vector, yielding plasmid pET28-σ^70^. *E. coli* strain BL21 (DE3) was transformed with expression plasmid pET28-σ^70^ and utilized for protein expression. The bacterial cells were grown in LB medium to the end of log phase and then a final concentration of 1 mM IPTG (isopropyl-β-D-thiogalactopyranoside) was added to the culture and the cells were harvested after incubation for another 4 h at 16°C. The cells were then harvested and disrupted by sonication on ice. The protein was purified from the supernatant with Ni^2+^-NTA agarose (Qiagen) according to the manufacturer's instructions.

### Electrophoretic mobility shift assay (EMSA)

For the electrophoretic mobility shift assay (EMSA), a 50 bp *nif* promoter fragment (from −47 to +3 relative to the transcription start site of *nifB* in *Paenibacillus* sp. WLY78) was synthesized by Sangon Biotech Co., Ltd (Shanghai). To do this, two DNA fragments corresponding to the sequences of the first strand (5′- GGAGAAGTGAATTGACTGTATTTGTCCCTGTCTCTAAGATGTAATTATAT-3′) and the complementary DNA strand (5′- ATATAATTACATCTTAGAGACAGGGACAAATACAGTCAATTCACTTCTCC-3′) were synthesized. The two strands were annealed and then labeled with digoxin using the DIG Gel Shift Kit (Roche). The binding of *E. coli* σ^70^-RNAP (RNA polymerase) (Epicentre) or σ^70^ of *Paenibaciillus* sp. WLY78 to the *nif* promoter was carried out using a gel shift kit (Roche). A scrambled 39 bp DNA fragment formed by annealing the following complementary oligonucleotides (5′- GTACGGAGTATCCAGCTCCGTAGCATGCAAATCCTCTGG-3′) and (5′-CCAGAGGATTTGCATGCTACGGAGCTGGATACTCCGTAC -3′) was used to assay non-specific binding.

To examine the specificity of binding to the promoter sequence *per se*, primers designed with substitutions in the −35 (TTGACT to GCTACT) and −10 (TAAGAT to GCAGAC) regions of the *nifB* promoter were utilized and were annealed and labeled as described above.

### DNAse I footprinting

The DNase I footprinting assay was performed as described by Zianni *et al.* [47]. A 365 bp *nif* promoter fragment (from −315 to +50 relative to the transcription start site) was PCR amplified from *Paenibacillus* sp. WLY78 with primer pfoot-up whose terminal base was fluorescent 6-carboxyfluorescein (FAM)-labeled and primer pfoot-down (Table S2). The 5′-FAM-labeled DNA fragment (400 ng) was incubated with the *E. coli* σ^70^-RNAP (10 pmol) for 30 min at 25°C. Bovine serum albumin (BSA) was used for the control experiment. After incubation, the mixtures were digested with DNase I for 40 seconds at 37°C and then the reactions were stopped by adding 0.2 M EDTA (pH 8.0). The digested DNA fragments were extracted with phenol-chloroform, precipitated with ethanol, and the pellets dissolved in Mini-Q water. The samples were sequenced with the ABI 3730 DNA analyzer by Genolab Co. and the data were analyzed with GeneMarker software.

### Construction of a *nifB* promoter::*lacZ* fusion

A 100 bp DNA fragment (P*nif*) (from −97 to +3 relative to the *nifB* transcription start codon) containing the *nifB* promoter was amplified from total DNA of *Paenibacillus* sp. WLY78 using primers (Table S2). The fragment was cloned into the promoterless plasmid pPR9TT, yielding plasmid pPR9TT-P*nif*. The plasmid was then transformed into *E. coli* JM109, yielding *E. coli*/P*nifB*::*lacZ*.

For β-galactosidase activity assays, *E. coli* JM109/pPR9TT and *E. coli*/P*nifB*::*lacZ* were grown overnight in LB medium at 30°C with shaking. The cultures were collected by centrifugation, washed three times with sterilized water and then resuspended in nitrogen-deficient medium containing 2 mM glutamate as nitrogen source to a final OD_600_ of 0.2–0.4. For measuring the effect of ammonium on nitrogenase activity, 1 ml culture was transferred to a 25 ml test tube supplemented with the concentration of NH_4_Cl indicated and the culture was incubated for 20 h at 30°C with shaking under anaerobic conditions. For measuring the effect of oxygen on nitrogenase activity, the test tubes were capped and filled with argon, and the oxygen concentration was adjusted to the initial concentration indicated and cultures were then incubated for 20 h at 30°C with shaking.

β-galactosidase activity was assayed according to the method described by Miller [48]. A 100 µl sample was taken and then mixed with 900 µl Z buffer containing β-mercaptoethanol, 40 µl chloroform and 20 µl 10% SDS and then shaken for 20 sec. Then 200 µl o-nitrophenyl-β-D-galactopyranoside (ONPG) (4 mg/ml) was added to the mixture and incubated in a water bath for 20 min at 28°C. The reaction was stopped with 500 µl 1M Na_2_CO_3_ solution. The mixture was then centrifuged for 15 min at 12000 rpm and the supernatant was used to measure the OD_420_ and OD_550_ values. 1 unit of β-galactosidase = [1000×(OD_420_−1.7 OD_550_)]/[Time (min)×vol (ml)×OD_600_].

### RT-PCR

For RT-PCR, *Paenibacillus* sp. WLY78 and the recombinant *E. coli* strains were grown in N_2_-fixing conditions (without NH_4_Cl and O_2_), non-N_2_-fixing conditions (100 mM NH_4_Cl and 21% O_2_) or at different concentrations of NH_4_Cl in the absence of O_2_ or at different concentration of O_2_ in the absence of NH_4_Cl. The cultures were harvested by centrifugation at 4°C, and total RNA was isolated using the PrimeScript RT reagent Kit with gDNA Eraser (Takara Bio) according to the manufacturer's instructions. The possibility of contamination of genomic DNA was eliminated by digestion with RNase-free DNase I (Takara Bio). The integrity and size distribution of the RNA was verified by agarose gel electrophoresis, and the concentration was determined spectrophotometrically. Synthesis of cDNA was carried out using RT Prime Mix according to the manufacturer's specifications (Takara Bio). 0.8 µg of cDNA was used for RT-PCR. The *nifH* and *nifK* transcripts were detected by using an RT-PCR Kit with 16S rDNA as a control. Primers for *nifH*, *nifK* and 16S rDNA used for PCR are listed in Table S2.

### Western blot assays for NifH and NifDK expression

For Western blotting, cultures of *Paenibacillus* sp. WLY78 and the engineered *E. coli* strains were grown either in non-N_2_-fixing conditions (LD medium and 21% O_2_) and harvested after 6–8 h of incubation or grown in N_2_-fixing conditions (2 mM glutamate and without O_2_) and harvested after 20 h of incubation, respectively. The cell pellet collected from 4 ml cultures at OD_600_ = 1 was dissolved in 200 µl sodium dodecyl sulfate (SDS) gel-loading buffer, boiled for 5 min and then 20 µl was loaded onto the stacking gel. Proteins were separated by sodium dodecyl sulfate polyacrylamide gel electrophoresis (SDS-PAGE) with an acrylamide∶bis-acrylamide ratio of 172∶1. Antisera raised against MoFe protein and Fe protein of *K. oxytoca* M5al were used as probes for Western blotting. The MoFe protein and Fe protein components of nitrogenase were purified from *K. oxytoca* M5al under anaerobic conditions and then used to make rabbit antiserum.

## Supporting Information

Figure S1 Immunological detection of nitrogenase MoFe protein and Fe protein in *Paenibacillus* sp. WLY78, the engineered *E. coli* strain 78-7 and *nif* gene deletion mutants. (**A**) Cultures grown in N_2_-fixing conditions (2 mM glutamate and in the absence of O_2_). (**B**) Cultures grown under non-N_2_-fixing conditions (LD medium and 21% O_2_). Antisera against *K. oxytoca* MoFe and Fe proteins, respectively, were used as probes. WT indicates *Paenibacillus* sp. WLY78. Vector indicates *E. coli* JM109 carrying empty vector pHY300PLK.
(TIF)

Table S1 Identity of *Paenibacillus* sp. WLY78 Nif polypeptides to those of other diazotrophs.
(DOC)

Table S2 Primers used in this study.
(DOCX)

Table S3 Bacterial strains and plasmids used in this study.
(DOCX)
