# Correction: A Minimal Nitrogen Fixation Gene Cluster from *Paenibacillus* sp. WLY78 Enables Expression of Active Nitrogenase in *Escherichia coli*

**DOI:** 10.1371/annotation/1e9bcb70-265a-4383-abf4-3466d144d56e

**Published:** 2013-10-29

**Authors:** Liying Wang, Lihong Zhang, Zhanzhi Liu, Dehua Zhao, Xiaomeng Liu, Bo Zhang, Jianbo Xie, Yuanyuan Hong, Pengfei Li, Sanfeng Chen, Ray Dixon, Jilun Li

The third author’s name was spelled incorrectly. The correct name is Zhanzhi Liu.

