# Supplementary figures and images for: A Minimal Nitrogen Fixation Gene Cluster from Paenibacillus sp. WLY78 Enables Expression of Active Nitrogenase in Escherichia coli
Source: PLoS Genet. 2013 Oct 17;9(10):e1003865. doi: 10.1371/journal.pgen.1003865 (PMC3798268; doi:10.1371/journal.pgen.1003865)

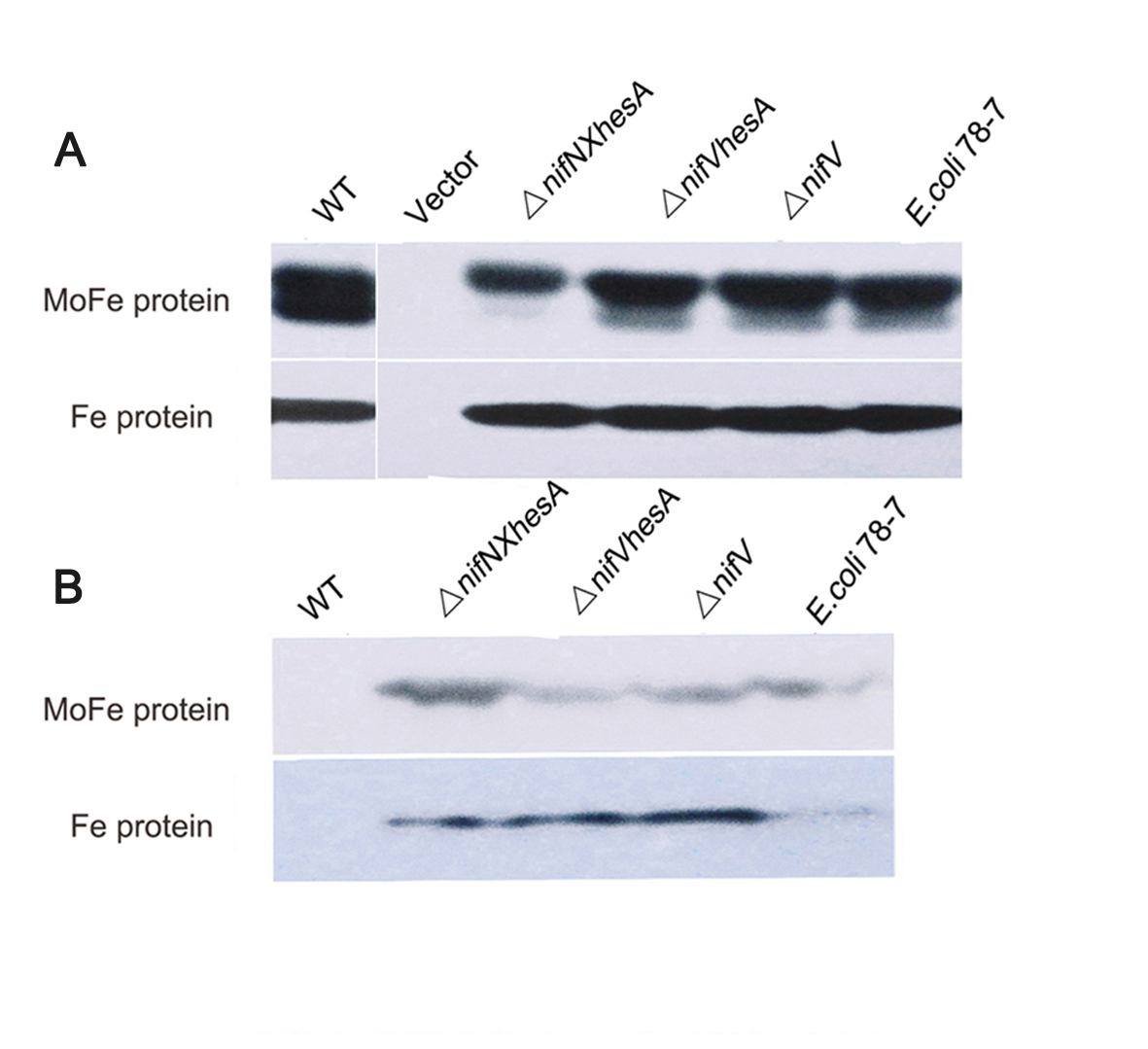

Supplement: Figure S1 — Immunological detection of nitrogenase MoFe protein and Fe protein in Paenibacillus sp. WLY78, the engineered E. coli strain 78-7 and nif gene deletion mutants. (A) Cultures grown in N2-fixing conditions (2 mM glutamate and in the absence of O2). (B) Cultures grown under non-N2-fixing conditions (LD medium and 21% O2). Antisera against K. oxytoca MoFe and Fe proteins, respectively, were used as probes. WT indicates Paenibacillus sp. WLY78. Vector indicates E. coli JM109 carrying empty vector pHY300PLK. (TIF) [file pgen.1003865.s001.tif]
