# Supplementary material for: A Minimal Nitrogen Fixation Gene Cluster from Paenibacillus sp. WLY78 Enables Expression of Active Nitrogenase in Escherichia coli
Source: PLoS Genet. 2013 Oct 17;9(10):e1003865. doi: 10.1371/journal.pgen.1003865 (PMC3798268; doi:10.1371/journal.pgen.1003865)
Supplement: Table S1 — Identity of Paenibacillus sp. WLY78 Nif polypeptides to those of other diazotrophs. (DOC) [file pgen.1003865.s002.doc]

| **Table S1.** Identity of *Paenibacillus* sp. WLY78 Nif polypeptides to those of other diazotrophs | | | | | | | | | | | |
| --- | --- | --- | --- | --- | --- | --- | --- | --- | --- | --- | --- |
|  | | | | | | | | | | | |
| **Gene** | ***nifB*** | ***nifH*** | ***nifD*** | ***nifK*** | ***nifE*** | ***nifN*** | ***nifX*** | ***hesA*** | ***nifV*** | **Accession number** |  |
| *Paenibacillus* sp. WLY78 | 100% | 100% | 100% | 100% | 100% | 100% | 100% | 100% | 100% | ALJV00000000 |  |
| *Paenibacillus massiliensis* T7 | 71.8% | 80.3% | 79.2% | 75.4% | 77.8% | 72.2% | 67.2% | - | - | AY912109 |  |
| *Clostridium acetobutylicum* | 34.8% | 54.1% | 51.8% | 47.8% | 44.7% | 37.5% | - | - | 37.9% | AE001437 |  |
| *Frankia* sp. EAN1pec | 60% | 28% | 32% | 53% | - | 90% | 98% | - | 72% | NC_009921 |  |
| *Methanococcus maripaludis* | - | 53.3% | 47.2% | 34.8% | 51.0% | 34.0% | 38.7% | - | - | |  | | --- |   NC_009135 |  |
| *Anabaena variablilis* ATCC29431 | 60% | 26% | - | 58% | - | 20% | - | - | 77% | NC_007413 |  |
| *K. oxytoca* | 37.1% | 69.7% | 64.0% | 50.7% | 55.7% | 47.4% | 43.6% | - | 48.7% | X13303 |  |
| *A. vinelandii* | 47.9% | 35.2% | 66.0% | 53.9% | 57.0% | 47.7% | 35.5% | - | 49.0% | M20568 |  |
