# Supplementary material for: A Minimal Nitrogen Fixation Gene Cluster from Paenibacillus sp. WLY78 Enables Expression of Active Nitrogenase in Escherichia coli
Source: PLoS Genet. 2013 Oct 17;9(10):e1003865. doi: 10.1371/journal.pgen.1003865 (PMC3798268; doi:10.1371/journal.pgen.1003865)
Supplement: Table S2 — Primers used in this study. (DOCX) [file pgen.1003865.s003.docx]

| **Table S2.** Primers used in this study | | | | | | |
| --- | --- | --- | --- | --- | --- | --- |
|  | | | | | | |
| **Gene name** | | **Forward primer (5' - 3')** | | **Reverse primer (5' - 3')** | | **Location /Target** |
| Complete *nif* cluster with *nif* promoter | S5.*ni f* cluster-up (GCTCTAGAGCGGAGACTATTTCCCAAAAT) | | S6.*nif* cluster-down  (TGCGGATCCGCGTGTAATGGTTATATGAAT) | | For cloning *nif* cluster to pHY300PLK | |
| Complete *nif* cluster without *nif* promoter | pET28-*nif*-up  (CGGGATCCAATGGACTCTTTAGCTGATCT) | | pET28*-nif*-down (CCCAAGCTTTAATGGTTATATGAATCAAGA) | | For cloning *nif* cluster to pET-28b | |
| *nifBHDKENXhesA* | S5*.nif* cluster-up (GCTCTAGAGCGGAGACTATTTCCCAAAAT) | | pS-V-down (CGCGGATCCGGTCATTAGCTTCATCTG) | | For construction of Δ*nifV* | |
| *nifBHDKENX* | S5*.nif* cluster-up (GCTCTAGAGCGGAGACTATTTCCCAAAAT) | | pS-O-down (GCGGATCCAACCATAACCGTAGCCTGCTTTA) | | For construction of Δ*hesAnifV* | |
| *nifBHDKEN* | S5*.nif* cluster-up (GCTCTAGAGCGGAGACTATTTCCCAAAAT) | | pS-X-down (GCGGATCCAACCATAACCGTAGCCTGCTTTA) | | For construction of Δ*nifXVhesA* | |
| *nifBHDKE* | S5*.nif* cluster-up (GCTCTAGAGCGGAGACTATTTCCCAAAAT) | | pS-N-down (CGCGGATCCGGTCATTAGCTTCATCTG) | | For construction of Δ*nifNXVhesA* | |
| *nifBHDKEN* | S5.*nif* cluster-up (GCTCTAGAGCGGAGACTATTTCCCAAAAT) | | *nifN*-XhoI (AACTCGAGCTTCATCCTCCCCTCCCCCTCTCG) | | For construction of Δ*nifX* or Δ*nifXhesA* | |
| *nifV* | *nifV*-UP-XS  (AACTCGAGAAGAGCTCGGAGGGGATACAGATGAGTCGG) | | S6.*nif* cluster-down  (TGCGGATCCGCGTGTAATGGTTATATGAAT) | | For construction of Δ*nifXhesA* or Δ*hesA* | |
| *nifBHDKENXhesA* | Orf1-up (AACTCGAGGGAGGGGATACAGATGCTGAGGAGGGCAGCGGC) | | S6.*nif* cluster-down  (TGCGGATCCGCGTGTAATGGTTATATGAAT) | | For construction of Δ*nifX* | |
| *nifBHDKENX* | S5.*nif* cluster-up (GCTCTAGAGCGGAGACTATTTCCCAAAAT) | | *nifX*-XhoI (AACTCGAGCTCATGATTCGGCTTTGCCGCTG) | | For construction of Δ*hesA* | |
| *nifX* | *nifX*-up-Hind (GCGAAGCTTATGAAGG TTGCATTTGCGACG) | | c-*nifX*-down  (TGGTCTAGACATGATTCGGCTTTGCCGCTG) | | For complement of Δ*nifX* | |
| *hesA* | *hesA*-up-Hind  (ATTAAGCTTATGCTGAGGAGGGCAGCGGC) | | *hesA*-down-XbaI  (GCGTCTAGACCGTCACGAAGTGTCGTATCAC) | | For complement of Δ*hesA* | |
| *nifXhesA* | *nifX*-up-Hind  (GCGAAGCTTATGAAGGTTGCATTTGCGACG) | | *hesA*-down-XbaI  (GCGTCTAGACCGTCACGAAGTGTCGTATCAC) | | For complement of Δ*nifXhesA* | |
| *nif* promoter | P*nifB-*100-up (CGGGGTACCCGT AAAATTTGACACATATG) | | P*nif*-down-Hind (GCCAAGCTTATATAATTAC ATCTTAGAGACAG) | | For construction of P*nif::lacZ* fusion | |
| *nif* promoter | pfoot-up (AGGAGAGCCGTATTTACGGAC) | | pfoot-down (GCTACTGGAAGATGAATGCGCG). | | For footprinting assay | |
| *rpoD* | Sigma A-F (CCGGAATTCGATGGCGAATGATCAGCATACT) | | Sigma A-R (ACGCGTCGACGTCCGCAATGTAACGCTTC-) | | σ^70^ | |
| *nifH* | pS-*nifH*-up (CGGGATCCTATGAGACAAATTGCGTTTTAC) | | *nifH*-R (ACCTGCCAGCTCTTCATACTC) | | RT-PCR | |
| *nifK* | *nifK*-up (ATGAGCGAGCGTCCGAATAT) | | *nifK*-down (TCAATGCCTCGGGAATTTC) | | RT-PCR | |
| 16S rDNA | *E. coli*-16S-F (GGTGATAGCGGTGAAATGCG) | | *E.coli*-16S-R (CTGGCAACAAAGGATAAGGGTT) | | RT-PCR (control) | |
| 16S rDNA | *Paeni*-16S-F (ATGGGCGAAGGAGGAAAGAC) | | *Paeni*-16S-R (CCAGGCGGAATGCTTARTGTG) | | RT-PCR (control) | |
| *nifB* | Random primer mix | | s6-GSP2 (ATCTCCAGGTCCCGCAATGCCG) | | 5’-RACE | |
| *nifB* | Random primer mix | | s6-GSP3 (TCATTGACGCAATCGAATTTG) | | 5’-RACE | |
