# Supplementary material for: A Minimal Nitrogen Fixation Gene Cluster from Paenibacillus sp. WLY78 Enables Expression of Active Nitrogenase in Escherichia coli
Source: PLoS Genet. 2013 Oct 17;9(10):e1003865. doi: 10.1371/journal.pgen.1003865 (PMC3798268; doi:10.1371/journal.pgen.1003865)
Supplement: Table S3 — Bacterial strains and plasmids used in this study. (DOCX) [file pgen.1003865.s004.docx]

| **Table S3.** Bacterial strains and plasmids used in this study | | | |
| --- | --- | --- | --- |
|  | | | |
| **Strain or plasmid** | **Relevant characteristic(s)** | **Reference or source** | |
| **Strains** |  |  | |
| *Paenibacillus* sp. WLY78 | Wild-type nitrogen fixer | | This lab. |
| *E. coli* strains |  | |  |
| JM109 | *recA1, endA1, gyrA96，thi-1，hsdR17, supE44, relA1,* Δ（*lac-proAB*）*/*F’[*traD36, proAB+, lacIq, lacZ*Δ*M15*] | | Sangon Biotech Co. |
| BL21 (DE3) | F^–^, *ompT, gal, dcm, lon, hsdSB*( *r_B_^-^m_B_^-^*-), λ (DE3 [*lacI, lacUV5-T7* gene 1, *ind1, sam7, nin5*]) | | Sangon Biotech Co. |
| JM109/pHY300PLK | Derivative of JM109, carrying empty vector pHY300PLK | | This study |
| 78-7 | Derivative of JM109, carrying plasmid pHY300-78 (P*nif*+*nif* cluster) which contains *nif* promoter and *nifBHDKENXVhesA* genes from *Paenibacillus* sp.WLY78; Amp^r^, Tet^r^ | | This study |
| 78-32 | Derivative of BL21 (DE3), carrying plasmid pET-32 which contains *nifBHDKENXVhesA* genes under control of T7 promoter; Kan^r^ | | This study |
| S-B (Δ*nifB*) | Derivative of BL21 (DE3), carrying pET-S-B (Δ*nifB*); Kan^r^ | | This study |
| S-V (*ΔnifV*) | Derivative of JM109, carrying plasmid pHY300-S-V (Δ*nifV*); Amp^r^, Tet^r^ | | This study |
| S-O (Δ*hesAnifV*) | Derivative of JM109, carrying plasmid pHY300-S-O; Amp^r^, Tet^r^ | | This study |
| S-X (Δ*nifXVhesA*) | Derivative of JM109, carrying plasmid pHY300-S-X (Δ*nifXVhesA*); Amp^r^, Tet^r^ | | This study |
| S-N (Δ*nifNXVhesA*) | Derivative of JM109, carrying plasmid pHY300-S-N (Δ*nifNXVhesA*); Amp^r^, Tet^r^ | | This study |
| D-XO (Δ*nifXhesA*) | Derivative of JM109, carrying plasmid pHY300-D-XO (Δ*nifXhesA*); Amp^r^, Tet^r^ | | This study |
| D-X (Δ*nifX*) | Derivative of JM109, carrying plasmid pHY300-D-X (Δ*nifX*); Amp^r^, Tet^r^ | | This study |
| D-O (Δ*hesA*) | Derivative of JM109, carrying plasmid pHY300-D-O (Δ*hesA*); Amp^r^, Tet^r^ | | This study |
| *E. coli*/P*nifB*::*lacZ* | Derivative of JM109; carrying plasmid P*nifB*::*lacZ* in which *nif* promoter is cloned in promoterless vector pPR9TT; Tet^r^, Cam^r^ | | This study |
| **Plasmids** |  | |  |
| pHY300PLK | A 4.87 kb shuttle vector between *B. subtilis* and *E. coli*; Amp^r^, Tet^r^ | | Sangon Biotech Co |
| pHY300PLK-78 | A 11 kb XbaⅠ-BamHⅠ DNA fragment including *nif* promoter and *nifBHDKENXVhesA* genes cloned to vector pHY300PLK; Amp^r^ , Tet^r^ | | This study |
| pHY300PLK-S-V | A 9.9 kb DNA fragment containing *nifBHDKENXhesA* cloned to vector pHY300PLK for constructing Δ*nifV* mutant; Amp^r^, Tet^r^ | | This study |
| pHY300PLK-S-O | A 9 kb DNA fragment containing *nifBHDKENX* cloned to vector pHY300PLK for constructing Δ*nifVhesA* mutant; Amp^r^, Tet^r^ | | This study |
| pHY300PLK-S-X | A 8.5 kb DNA fragment containing *nifBHDKEN* cloned to vector pHY300PLK for constructing Δ*nifXVhesA* mutant; Amp^r^, Tet^r^ | | This study |
| pHY300PLK-S-N | A 7.37 kb DNA fragment containing *nifBHDKE* cloned to vector pHY300PLK for constructing Δ*nifNXVhesA* mutant; Amp^r^, Tet^r^ | | This study |
| pHY300PLK-D-XO | A 8444 bp *nifBHDKEN* DNA fragment and a 1331 bp *nifV* DNA fragment were ligated together, and then cloned to vector pHY300PLK for constructing Δ*nifXhesA* mutant; Amp^r^, Tet^r^ | | This study |
| pHY300PLK-D-X | A 8444 bp *nifBHDKEN* DNA fragment and a 2202 bp *nifVhesA* DNA fragment were ligated together, and then cloned to vector pHY300PLK for constructing Δ*nifX* mutant; Amp^r^, Tet^r^ | | This study |
| pHY300PLK-D-O | A 8870 bp *nifBHDKENX* DNA fragment and a 1331 bp *nifV* DNA fragment were ligated together, and then cloned to vector pHY300PLK for constructing Δ*hesA* mutant; Amp^r^, Tet^r^ | | This study |
| pET-28b | Expression vector, T7 promoter and T7 terminator; pBR322 origin; Kan^r^ | | Sangon Biotech Co |
| pET-78-32 | A 10.7 kb DNA fragment containing a complete *nif* cluster including *nifBHDKENXVhesA* without *nif* promoter; Kan^r^ | | This study |
| pET-S-B | A 10.2 kb DNA fragment containing *nifHDKENXVhesA* cloned to pET-28b; Kan^r^ | | This study |
| pPR9TT | Broad-host range *lacZ* promoter probe vector; RK2 replicon; Amp^r^, Cam^r^ | | Sangon Biotech Co |
| P*nifB*::*lacZ* | A 100 bp *nif* promoter fragment cloned to promoterless vector pPR9TT which is a *lacZ* probe vector, Amp^r^, Cam^r^ | | This study |
